# Natural compound Alternol actives multiple endoplasmic reticulum stress-responding pathways contributing to cell death

**DOI:** 10.3389/fphar.2024.1397116

**Published:** 2024-05-20

**Authors:** Wang Liu, Chenchen He, Changlin Li, Shazhou Ye, Jiang Zhao, Cunle Zhu, Xiangwei Wang, Qi Ma, Benyi Li

**Affiliations:** ^1^ Department of Urology, The University of Kansas Medical Center, Kansas City, KS, United States; ^2^ Department of Radiation Oncology, The First Affiliated Hospital of Xi’an Jiaotong University School of Medicine, Xi’an, China; ^3^ Tianjin Institute of Urology, The Second Hospital of Tianjin Medical University, Tianjin, China; ^4^ Translational Research Laboratory for Urology, The First Affiliated Hospital of Ningbo University, Ningbo, Zhejiang, China; ^5^ Department of Urology, The Affiliated Hospital of Guangdong Medical University, Zhanjiang, China

**Keywords:** Er stress, PERK, IRE1α, prostate cancer, heat-shock proteins, ATP release

## Abstract

**Background:** Alternol is a small molecular compound isolated from the fermentation of a mutant fungus obtained from Taxus brevifolia bark. Our previous studies showed that Alternol treatment induced reactive oxygen species (ROS)-dependent immunogenic cell death. This study conducted a comprehensive investigation to explore the mechanisms involved in Alternol-induced immunogenic cell death.

**Methods:** Prostate cancer PC-3, C4-2, and 22RV1 were used in this study. Alternol interaction with heat shock proteins (HSP) was determined using CETSA assay. Alternol-regulated ER stress proteins were assessed with Western blot assay. Extracellular adenosine triphosphate (ATP) was measured using ATPlite Luminescence Assay System.

**Results:** Our results showed that Alternol interacted with multiple cellular chaperone proteins and increased their expression levels, including endoplasmic reticulum (ER) chaperone hypoxia up-regulated 1 (HYOU1) and heat shock protein 90 alpha family class B member 1 (HSP90AB1), as well as cytosolic chaperone heat shock protein family A member 8 (HSPA8). These data represented a potential cause of unfolded protein response (UPR) after Alternol treatment. Further investigation revealed that Alternol treatment triggered ROS-dependent (ER) stress responses via R-like ER kinase (PERK), inositol-requiring enzyme 1α (IRE1α). The double-stranded RNA-dependent protein kinase (PKR) but not activating transcription factor 6 (ATF6) cascades, leading to ATF-3/ATF-4 activation, C/EBP-homologous protein (CHOP) overexpression, and X-box binding protein XBP1 splicing induction. In addition, inhibition of these ER stress responses cascades blunted Alternol-induced extracellular adenosine triphosphate (ATP) release, one of the classical hallmarks of immunogenic cell death.

**Conclusion: **Taken together, our data demonstrate that Alternol treatment triggered multiple ER stress cascades, leading to immunogenic cell death.

## Introduction

ER is a crucial organelle with a lot of functions, including storage and buffering of calcium ions (Ca^2+^), lipid biosynthesis, and folding and assembly of secretory and transmembrane proteins (Celik et al., 2023). However, due to physical and chemical factors, cell homeostasis is easy to be destroyed and cellular proteins cannot be properly folded, causing a series of physiological responses, including lack of Ca^2+^ deficiency, molecular chaperone or cellular energy, and increased reactive oxygen species (ROS), protein variation and disulfide bond reduction (Oakes and Papa, 2015; Rufo et al., 2017). For maintaining ER homeostasis, cells have developed an adaptation mechanism through a series of adaptive pathways called the unfolded protein response (UPR) (Hetz et al., 2020).

The UPR aims to recover the ER-related protein folding ability by increasing the expression of ER-related chaperones and attenuating global protein translation (Rufo et al., 2017). In mammalian cells, the UPR is controlled by three ER stress sensors, namely, IRE1α, PERK, and activating transcription factor 6 (ATF6). In homeostasis conditions, these proteins are kept in an inactive state by the master regulator of the UPR, the glucose-regulated protein-78 (GRP78, also known as BiP) (Ernst et al., 2024). PERK protein responds to ER stress by inducing eIF2α phosphorylation, resulting in increased expression of ATF4 protein and CHOP (Saaoud et al., 2024). IRE1α protein cleaves the transcription factor X-box binding protein (XBP1) mRNA to generate a spliced XBP1 variant (XBP1s), which triggers ER stress by up-regulating a large number of genes involved in the UPR (Park et al., 2021). During ER stress, the ATF6 protein is translocated from the ER to the Golgi apparatus for cleavage by S1P and S2P. The cleaved N-terminal region of ATF6 protein is an active transcription factor for ER chaperones and XBP1 (de la Calle et al., 2022).

Immunogenic cell death (ICD) is a subtype of cell death that triggers an adaptive immune response against remaining tumor cells (Aria and Rezaei, 2023; Sprooten et al., 2023). Certain chemo-drugs, radiation therapy, and photodynamic therapy were reported to induce ICD on treated tumor cells by eliciting ER stress and subsequent secretion of damage-associated molecular patterns (DAMPs), including calreticulin membrane translocation, ATP and HMGB1 release, type-I interferon production, etc. (Galluzzi et al., 2020). These DAMPs then recruit innate immune cells such as dendritic cells to stimulate tumor-specific cytotoxic T lymphocytes to eliminate remaining tumors. We recently demonstrated that the chemo-drug Mitoxantrone was a *bona-fade* ICD inducer for prostate cancer by activating eIF2α *via* PERK/GCN2-dependent ER stress cascade (Li et al., 2020). We also discovered that Alternol, a novel small chemical compound, induced a strong ICD response in prostate cancer *via* releasing large amounts of inflammatory cytokines while the molecular mechanism was not determined (Li et al., 2021).

Alternol was isolated from the fermentation of a mutant fungus obtained from *Taxus brevifolia* bark (Liu et al., 2020). Previous studies from our group and others demonstrated that Alternol treatment in prostate cancer cells caused a dramatic increase in reactive oxygen species and subsequent cell death (Tang et al., 2014; Zuo et al., 2017; Xu et al., 2019; Xu et al., 2020). We also discovered that Alternol interacted with 14 cellular proteins including five mitochondrial and ER-residing chaperone proteins, indicating a potential link between Alternol-induced ICD (inflammatory response) (Li et al., 2021) and ER stress. To determine if Alternol-induced ICD responses were due to chaperone protein disruption and ER stress, we conducted a series of experiments to investigate ER stress-related cascades in Alternol-treatment prostate cancer cells. Our data confirmed that Alternol treatment elicited multiple ER stress cascades and subsequent immunogenic ATP release.

## Materials and methods

### Cell lines, reagents, antibodies, and siRNA

Human prostate cancer C4-2B, 22RV1, PC-3 cells, and Benign Prostatic Hyperplasia-1 (BPH1) cells were recently obtained from ATCC (Manassas, VA) and authenticated by ATCC before shipment. Cells were cultured in RPMI1640 medium (Gibco) supplemented with 10% fetal bovine serum (FBS, Gibco) plus 100 U/mL penicillin/streptomycin and 2 mM L-glutamine.

Antibodies of ATF-3 (#33593), ATF-6 (#65880), IRE1α (#3284), ATF4 (#11815), eIF2α (#9722), eIF2α/pS51 (#9721), CHOP (#2895), BiP (#3177), XBP1s (#27091), PERK (#5683), phospho-PERK (T980, #3179), PARP (#9542), HSP60 (D6F1, #12165), HSPA8 (#8444), HYOU1 (#13452), NF-κB/p65 (#3034), phospho-NF-κB (S563, #3031), I-κBa (#4024), and IKK (#2684) were obtained from Cell Signaling (Danvers, MA, United States of America). Antibodies of HSP90B1 (#H9010) and phospho-IRE1α (Ser724) (# PA1-16927) were purchased from Thermo Fisher (Waltham, MA, United States of America). Antibodies of HSP90A1 (sc-13119) and β-Actin (sc-47778) were obtained from Santa Cruz (Dallas, TX, United States of America).

Alternol was obtained as a gift from Sungen Biosciences (Shantou, China). PERK inhibitor AMG44 (#SML3049), and ATF-6 inhibitor CEAPIN-A7 (SML2330) were obtained from Millipore Sigma (Burlington, MA, United States of America). PKR inhibitor Imoxin (S9668), NF-κB inhibitor SN50 (S6672), and IRE1α inhibitor MKC8866 (S8875) were obtained from Sellectchem (Huston, TX, United States of America). ROS scavenger n-acetylcysteine (N-Ac) was obtained from Cayman Chemicals (Ann Arbor, MI). The small interfering RNAs (siRNAs) for IRE1a, PERK, and PKR were obtained from Horizon Discovery Ltd (Cambridge, UK). ATPlite™ luminescence assay system (catalog #6016941) was purchased from PerkinElmer (Waltham, MA).

### Western blot assays

Western blot assay of protein expression was conducted as described (Li et al., 2019). Briefly, total cellular protein lysates were extracted using the radioimmunoprecipitation assay (RIPA) buffer supplemented with a protease inhibitor cocktail. An equal amount of proteins was subjected to SDS-PAGE separation, followed by transferring onto the PVDF membrane. After blocking in 5% nonfat milk for 1h, the membranes were incubated with primary antibodies overnight at 4°C. Protein bands were visualized using the horseradish peroxidase-linked (HRP-linked) secondary antibody for 2 h and the ECL solution (Santa Cruz Biotech).

### ATP level assay

ATP level was measured using ATPlite™ Luminescence Assay System following the manufacturer’s instructions, as described (Li et al., 2021). Briefly, C4-2B or PC-3 cells were seeded in a 6-well plate overnight, and then treated as described. The cell pellets were collected and lysed in RIPA buffer. The cellular lysate was then diluted with the assay buffer, and mixed with the substrate solution (at a 4:1 ratio). The luminescence signal was measured using the Lumat LB9501 reader (Berthold, Oak Ridge, TN).

### Cellular thermal shift assay (CETSA)

Alternol binding with the chaperone proteins was examined using the CETSA assay, as described previously (He et al., 2017). Briefly, C4-2B cells were incubated with Alternol (10 μM) for 2 h. Cell pellets were washed with PBS followed by two repeated freeze-thaw cycles with liquid nitrogen. The lysates were then aliquoted into 9 vials for heating at 37, 41, 45, 49, 53, 57, 61, 65, and 69°C for 3 min, and then cooled down on ice for 2 min. The cell lysates were briefly vortexed and then centrifuged at 18,000 g for 20 min at 4°C. The supernatant was loaded onto SDS-PAGE gel followed by Western blot analysis.

### Statistical analysis

Data were present as the mean ± SEM from at least three experiments. Representative images of non-quantitative data were shown from multiple experiments. Statistical analysis was conducted using ANOVA analysis and student *t*-test to compare two groups with SPSS software (Chicago, IL). A *p*-value of 0.05 or less was considered as a significant difference.

## Results

### Alternol interacts with multiple heat-shock proteins

**FIGURE 1:**
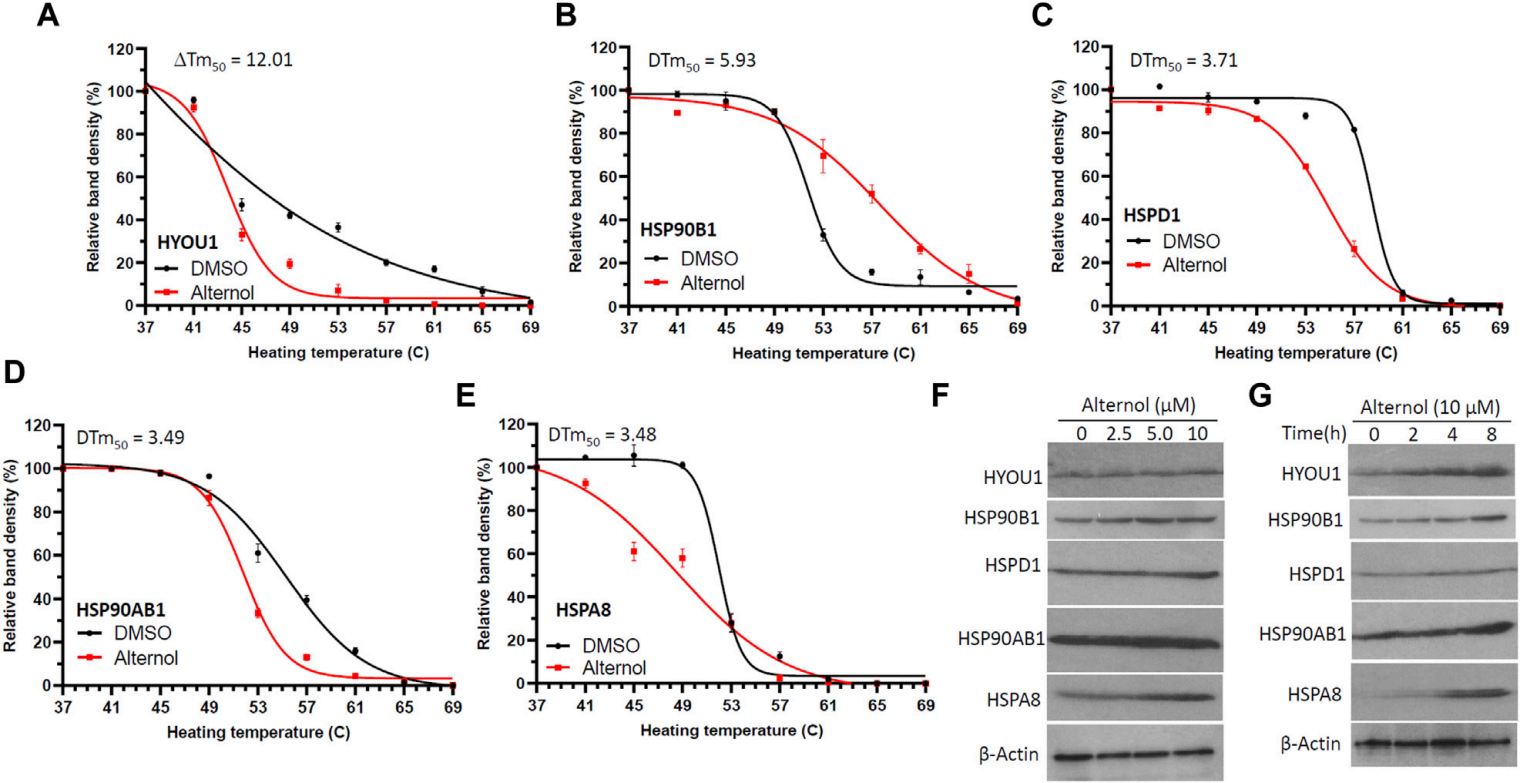
Alternol interacts with multiple chaperone proteins. **(A–E)** C4-2B cells were treated with Alternol (10 μM) for 2 h. Cell pellets were washed with cold PBS, and then lysed through two freeze-thaw cycles in liquid nitrogen. Cell lysates were aliquoted to 9 tubes and then heated at 37, 41, 45, 49, 53, 57, 61, 65, and 69°C for 3 min. After colling down on the ice for 2 min, the lysates were centrifuged for 20 min. The supernatants were collected for Western blot analysis. Average band density from three independent experiments was used for the curve fitting analysis as described previously in our publication (Li et al., 2019). **(F,G)** C4-2B cells were treated with Alternol in different concentrations (0, 2.5, 5, 10 μM) or periods (0, 2, 4, 8 h) at 10 μM. Cells were collected for Western blot analysis. β-Actin blot was used as the protein loading control.

We and others have shown that Alternol induces oxidative stress-dependent cell death preferentially in malignant cells (Tang et al., 2014; Zuo et al., 2017; Xu et al., 2019; Liu et al., 2020; Xu et al., 2020). In our previous report (Li et al., 2019), to identify Alternol-interacting proteins we utilized biotin-labeled Alternol and pulled down 14 cellular proteins. Among these proteins, there were five chaperone proteins, including two ER-residing HYOU1 (also known as HSP120α or GRP170) and HSP90B1 (or GRP94), mitochondrial HSPD1 (or HSP60), cytosolic HSP90AB1 (or HSP84) and heat-shock cognate HSPA8 (HSC70). To further verify their interaction with Alternol, we conducted a CETSA assay (Martinez Molina et al., 2013) in prostate cancer PC-3 cells. As shown in Figures 1A–E, these proteins displayed a clear curve-shifting pattern after Alternol treatment with a ΔT_m_50 value between 3.48C and 12.01C. These data indicated that Alternol interacted with these proteins in cells. In addition, Alternol treatment increased the expression levels of HYOU1, HSP90B1, HSP90AB1, and HSPA8 proteins (Figures 3F, G). Since these chaperone proteins are involved in protein folding and ER stress protection (Lindenmeyer et al., 2008; Rachidi et al., 2015; Lin et al., 2023; Tao et al., 2024), these data indicate that Alternol treatment potentially caused an unfolded protein response (UPR) and subsequent ER stress.

### Alternol induces ER stress responses via a ROS-dependent mechanism

**FIGURE 2:**
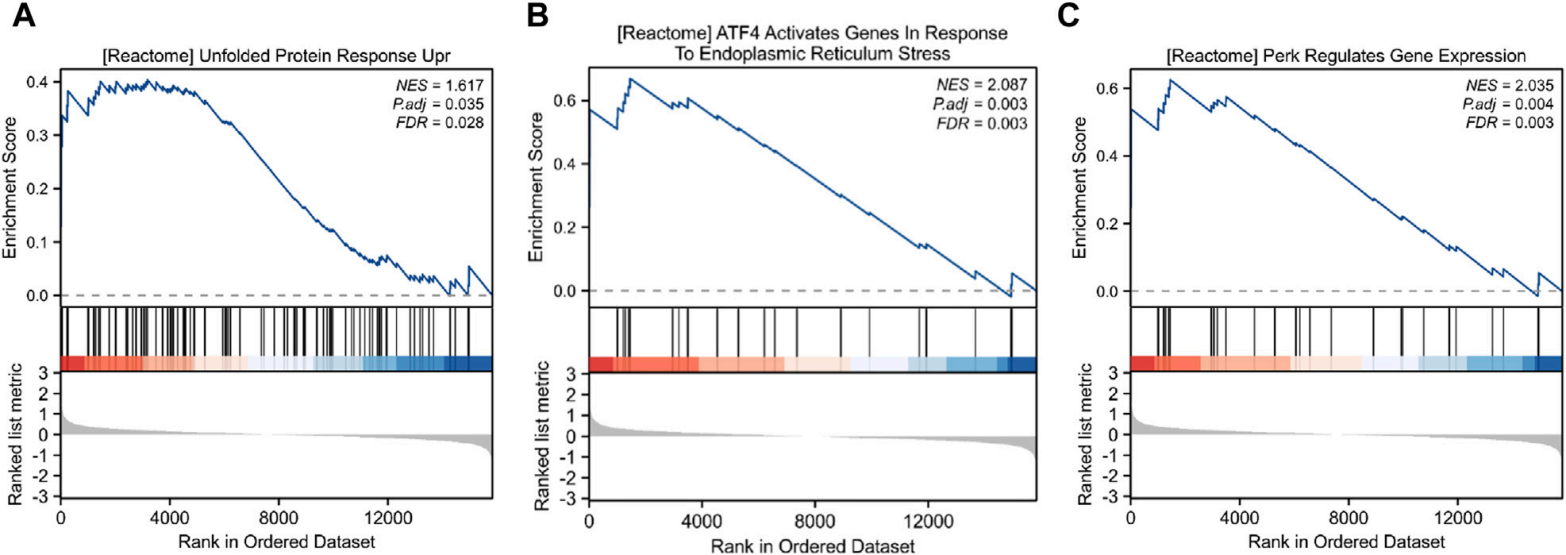
GSEA analysis of RNA-seq data for gene expression alterations after Alternol treatment. Activation of the unfolded protein response **(A)**, ATF4 **(B)**, and PERK **(C)** pathways was noticed in PC-3 cells after Alternol treatment.

To elucidate the detail of Alternol-induced UPR and ER stress response, we re-analyzed the RNA-seq data generated from Alternol-treated PC-3 cells as described in our recent publication (Li et al., 2021). As shown in Table 1, the most upregulated genes after Alternol treatment were inflammatory cytokines and ER stress responding factors, including CXCL8, IL1A, IL6, CXCL3, CCL20, CXCL2, DDIT3 (CHOP), IL1B, and ATF3. Gene set enrichment analysis (GSEA) revealed that unfolded protein response (UPR) and PERK/ATF4-related ER stress response pathways were highly activated after Alternol treatment (Figure 2; Table 2). These data strongly suggest that Alternol treatment caused UPR and ER stress.

**TABLE 1:** The most significantly upregulated genes after Alternol treatment.

Symbol	log2FC	P adj	functional significance
**CXCL8**	3.069614276	8.25E-148	chemotactic factor for the neutrophils to infection site and a potent angiogenic factor
**CYP1A1**	2.989899427	4.64E-29	ER protein for PAHs metabolism to carcinogens
**IL1A**	2.903908725	3.17E-26	A pleiotropic cytokine produced by monocytes and macrophages in response to cell injury and involved in immune responses, inflammatory processes and hematopoiesis
**FGF21**	2.818916616	1.30E-27	A secreted metabolic regulator stimulating the uptake of glucose in adipose tissue
**IL6**	2.524239099	7.52E-137	A cytokine in inflammation and the maturation of B cells
**CLDN1**	2.07557688	1.09E-15	An integral membrane protein and a component of tight junction strands
**CXCL3**	2.060143538	4.46E-15	A secreted CXCR2 ligand for inflammation and a chemoattractant for neutrophils
**SPOCD1**	2.033185799	1.45E-18	A TFIIS family protein of transcription elongation factor
**EREG**	2.030978558	8.37E-40	A secreted EGF family protein structurally related to ERBB4 and involved in inflammation, wound healing, oocyte maturation, and cell proliferation
**CCL20**	1.988531829	2.44E-06	small chemotactic antimicrobial cytokine CC gene for lymphocytes but repressing proliferation of myeloid progenitors
**CXCL2**	1.984503994	4.12E-37	a CXC subfamily antimicrobial secreted protein expressed at sites of inflammation suppressing hematopoietic progenitor cell proliferation
**MMP3**	1.856126057	6.01E-05	an enzyme for fibronectin, laminin, collagens III, IV, IX, and X, and cartilage proteoglycans degradation invovled in wound repair, progression of atherosclerosis and tumor initiation
**INHBE**	1.837234608	6.66E-25	TGF-beta superfamily regulating cell proliferation, apoptosis, immune response and hormone secretion under ER stress
**DDIT3**	1.823943929	4.86E-83	C/EBPzeta or CHOP activated after ER stress for apoptosis
**TMEM191C**	1.785536372	1.17E-04	transmembrane protein
**IL1B**	1.760248754	1.57E-04	produced by macrophages and processed CASP1 involved in cell proliferation, differentiation, and apoptosis
**DHRS9**	1.753826235	1.75E-04	a moonlighting protein in short-chain dehydrogenases-reductases (SDR) family
**ATF3**	1.751932311	9.22E-74	a CREB protein family involved in the complex process of cellular stress response
**IER3**	1.734525965	4.10E-239	protection of cells from Fas- or TNF alpha-induced apoptosis
**PNPLA1**	1.698159315	4.79E-10	patatin-like phospholipase family protein in lipid and glycerophospholipid metabolism
**PHLDA1**	1.692770358	3.85E-131	a proline-histidine rich nuclear protein in the anti-apoptotic effects of IGF-1
**DUSP1**	1.647561862	1.99E-102	a phosphatase with dual specificity for tyrosine and threonine of ERK2 in cellular sress and negative regulation of cellular proliferation
**CPA4**	1.643863159	1.37E-17	a carboxypeptidase A/B subfamily protein involved in histone hyperacetylation pathway
**SCN4A**	1.600392345	2.42E-14	sodium voltage-gated channel alpha subunit 4 Nav1.4
**C11orf96**	1.579647153	1.64E-05	1242 nt
**ARHGAP9**	1.566966431	9.75E-04	Rho GAP9 towards Rho-family GTPases *in vitro* regulating adhesion of hematopoietic cells to the extracellular matrix
**DPP4**	1.566467628	1.57E-10	CD26, an intrinsic membrane glycoprotein and a serine exopeptidase that cleaves X-proline dipeptides from the N-terminus of polypeptides
**GLIPR1**	1.566363867	4.99E-39	decreased expression of this gene through gene methylation is associated with prostate cancer hodling proapoptotic activities in prostate and bladder cancer cells
**PSG1**	1.564068691	1.00E-03	a major product of the syncytiotrophoblast in placenta
**PPP1R15A**	1.562368114	5.17E-72	proapoptotic protein induced by stressful growth arrest conditions and treatment with DNA-damaging agents regardless of p53 status
**HIST1H1E**	1.558191745	9.08E-05	a linker histone H1 interacts with linker DNA between nucleosomes H1.4
**GDF15**	1.544564896	6.35E-72	a TGF-beta family ligand in activation of SMAD transcription factors involved in the stress response of hypoxia, inflammation, acute injury and oxidative stress
**MPZ**	1.538122369	6.18E-04	a type I transmembrane glycoprotein specifically expressed in Schwann cells of the peripheral nervous system that is a major structural protein of the peripheral myelin sheath
**PSG4**	1.524354694	2.61E-31	a member of the carcinoembryonic antigen (CEA) gene family and may play a role in regulation of the innate immune system
**HIST1H1D**	1.521422522	1.62E-04	H1D-H1.3
**MAGEC2**	1.507672547	1.47E-03	expressed only in tumors of various histological types
**AREG**	1.503432175	3.00E-18	an EGF member autocrine and mitogen interacts with EGFR/TGF-a receptor to promote the growth of epithelial cells but inhibits aggressive carcinoma cell lines

**TABLE 2:** GSEA enrichment of ER stress and inflammatory pathways after Alternol treatment.

Pathway ID and description	setSize	enrichmentScore	NES	P-value	p.adjust	qvalue	core_enrichment
REACTOME_UNFOLDED_PROTEIN_RESPONSE_UPR	90	0.403810332095922	1.61739253010353	0.00371964709976059	0.0347455407380737	0.0278176790447212	CXCL8/DDIT3/ATF3/CREB3L3/CREB3L1/ERN1/CEBPB/ASNS/EXOSC8/EXOSC7/EXOSC1/EXOSC3/CREB3/DNAJB9/LMNA/ATP6V0D1/SRPRB/ZBTB17/GFPT1/EXOSC2/EIF2S1/XBP1/EXOSC9
REACTOME_ATF4_ACTIVATES_GENES_IN_RESPONSE_TO_ENDOPLASMIC_RETICULUM_STRESS	26	0.669544754867975	2.08679020953492	0.000136088569553141	0.00324726190715911	0.00259979518489743	CXCL8/DDIT3/ATF3/CEBPB/ASNS/EXOSC8/EXOSC7/EXOSC1/EXOSC3
REACTOME_PERK_REGULATES_GENE_EXPRESSION	31	0.624849485874999	2.03523436113533	0.000185971399602142	0.00409290845155759	0.00327682952247437	CXCL8/DDIT3/ATF3/CEBPB/ASNS/EXOSC8/EXOSC7/EXOSC1/EXOSC3
REACTOME_RESPONSE_OF_EIF2AK1_HRI_TO_HEME_DEFICIENCY	15	0.831017551110207	2.25397975547525	1.73707362397832E-06	0.00016745389735151	0.000134065513800095	DDIT3/ATF3/PPP1R15A/CHAC1/TRIB3/ATF5/CEBPB/ASNS
WP_OVERVIEW_OF_PROINFLAMMATORY_AND_PROFIBROTIC_MEDIATORS	69	0.666293872828877	2.54058809095234	1E-10	0.0000000482	3.85894736842105E-08	CXCL8/IL1A/IL6/CXCL3/CCL20/CXCL2/MMP3/IL1B/AREG/CCL26/MMP1/LIF/CCL24/IL11
WP_PHOTODYNAMIC_THERAPYINDUCED_NFKB_SURVIVAL_SIGNALING	31	0.769362889109306	2.50593754735177	5.27116095919563E-09	2.11724965194358E-06	1.69509439266765E-06	CXCL8/IL1A/IL6/CXCL2/MMP3/IL1B/MMP1/BIRC3/CCND1/NFKB2/ICAM1
REACTOME_INTERLEUKIN_10_SIGNALING	36	0.728492156095692	2.41319894329377	3.77827702097557E-08	7.49878624272726E-06	6.00361440616688E-06	CXCL8/IL1A/IL6/CCL20/CXCL2/IL1B/LIF
WP_LTF_DANGER_SIGNAL_RESPONSE_PATHWAY	16	0.828588190440065	2.29128292141293	3.93571600941655E-06	0.000271002159505539	0.000216967441962723	CXCL8/IL1A/IL6/IL1B
REACTOME_DECTIN_1_MEDIATED_NONCANONICAL_NF_KB_SIGNALING	59	0.591165749821468	2.20455108416999	2.24146074500624E-06	0.000200071125757964	0.000160179241543721	MAP3K14/PSMD6/PSMC1/PSMD13/PSMD14/PSMB6/PSMC4/PSMD8/PSMC2/NFKB2/PSMB7/PSMB3/PSMB1/PSMB4/PSMB5/PSMA7/PSME3/PSMD12/PSMD2/PSMA1/PSMC5/PSMD4/PSMD3/PSMA5/UBE2M/PSMA3/PSMD11/PSMB2/PSMF1/PSMA2/PSMC3/PSMC6/PSMA4/PSMD7
REACTOME_TNFR2_NON_CANONICAL_NF_KB_PATHWAY	94	0.469873707004391	1.88843053915875	0.000112012261310524	0.00293423423650394	0.00234918163595183	TNFRSF12A/TNFSF18/TNFRSF9/BIRC3/MAP3K14/PSMD6/PSMC1/PSMD13/PSMD14/PSMB6/PSMC4/PSMD8/PSMC2/NFKB2/PSMB7/LTBR/PSMB3/PSMB1/PSMB4/PSMB5/PSMA7/PSME3/PSMD12/PSMD2/PSMA1/PSMC5/PSMD4/PSMD3/PSMA5/UBE2M/PSMA3/PSMD11/PSMB2/PSMF1/PSMA2/PSMC3/PSMC6/TNFRSF1A/PSMA4
REACTOME_SIGNALING_BY_INTERLEUKINS	404	0.372894448305077	1.82136922961858	4.35614138581666E-08	7.49878624272726E-06	6.00361440616688E-06	CXCL8/IL1A/IL6/CCL20/CXCL2/MMP3/IL1B/IL7R/MEF2C/SERPINB2/STAT4/IL2RB/MMP1/HMOX1/LIF/CDKN1A/MAOA/IL12RB1/CD36/IRAK2/CSF1R/ANXA1/IL11/PSMD6/DUSP4/SNRPA1/PSMC1/JUN/GAB2/HNRNPDL/PSMD13/FOXO1/DUSP6/PSMD14/PSMB6/PSMC4/IL21R/SOCS1/PSMD8/PSMC2/BOLA2B/BOLA2/S1PR1/CCND1/HAVCR2/NFKB2/NFKBIB/PSMB7/ICAM1/ANXA2/PSMB3/PSMB1/PSMB4/S100A12/TGFB1/PSMB5/PSMA7/LCN2/PSME3/PSMD12/PSMD2/JAK1/PSMA1/ITGAX/VEGFA/ARF1/SOD1/PSMC5/ITGAM/PSMD4/PTPN2/SQSTM1/PSMD3/IL16/PSMA5/CSF3/MAP2K3/EBI3/PSMA3/STX1A/PSMD11/IL13RA2/PSMB2/PSMF1/PSMA2/IL15/CNN2/PSMC3/PSMC6/MAPK10/PPP2R1A/CD80
WP_INTERLEUKIN1_INDUCED_ACTIVATION_OF_NFKB	10	0.670824274298685	1.60513022644812	0.0212616867687299	0.118612650723702	0.0949626506995174	IL1A/AJUBA/SQSTM1
REACTOME_INTERLEUKIN_1_FAMILY_SIGNALING	133	0.432313543114489	1.83653655910291	3.613182646093E-05	0.00126979828293217	0.00101661509177317	IL1A/IL1B/IRAK2/PSMD6/PSMC1/PSMD13/PSMD14/PSMB6/PSMC4/PSMD8/PSMC2/NFKB2/NFKBIB/PSMB7/PSMB3/PSMB1/PSMB4/S100A12/PSMB5/PSMA7/PSME3/PSMD12/PSMD2/PSMA1/PSMC5/PSMD4/PTPN2/SQSTM1/PSMD3/PSMA5/PSMA3/PSMD11/PSMB2/PSMF1/PSMA2/PSMC3/PSMC6
REACTOME_INTERLEUKIN_4_AND_INTERLEUKIN_13_SIGNALING	96	0.465496512696374	1.87979168950261	8.53180753047583E-05	0.00232828257097992	0.00186404977183061	CXCL8/IL1A/IL6/MMP3/IL1B/MMP1/HMOX1/LIF/CDKN1A/MAOA/CD36/ANXA1/FOXO1/SOCS1/S1PR1/CCND1/ICAM1/TGFB1/LCN2/JAK1/ITGAX/VEGFA/ITGAM
BIOCARTA_IL10_PATHWAY	12	0.8339679706654	2.13295111435416	2.7558613693034E-05	0.00107122998387439	0.00085763903054892	IL1A/IL6/HMOX1/BLVRB
WP_IL10_ANTIINFLAMMATORY_SIGNALING_PATHWAY	11	0.844548136912348	2.07961312966775	6.50967140375665E-05	0.00189015760036789	0.00151328188751883	IL1A/IL6/HMOX1/BLVRB
REACTOME_INTERLEUKIN_12_FAMILY_SIGNALING	54	0.465672585039885	1.7178504278712	0.00347140036911967	0.0331987098792794	0.0265792684903774	SERPINB2/STAT4/IL12RB1/SNRPA1/HNRNPDL/BOLA2B/BOLA2/ANXA2/JAK1/ARF1/SOD1/EBI3/CNN2/TCP1
BIOCARTA_IL17_PATHWAY	10	0.757360892312669	1.81219271149915	0.00265695294069546	0.0273643443892139	0.021908208458366	CXCL8/IL6
WP_IL18_SIGNALING_PATHWAY	242	0.426822735994919	1.96172264663375	1.99205143391354E-08	4.80084395573163E-06	3.84361081933002E-06	CXCL8/IL6/CLDN1/CXCL3/CCL20/CXCL2/MMP3/IL1B/ATF3/IER3/PTX3/MMP1/HMOX1/BMP2/TGM2/ARL4D/CD36/FUT1/BIRC3/RUNX2/IRF1/EPS8/NR1H3/JUN/TOMM40/SDC4/TNFAIP3/ZC3H12A/RND2/BID/FAM186B/RAE1/NFKB2/CEBPB/ICAM1
PID_IL23_PATHWAY	29	0.589289764820712	1.87852782402297	0.00156416869931681	0.0192328906395588	0.0153980731785592	IL6/IL1B/STAT4/IL12RB1
PID_IL27_PATHWAY	23	0.612243789203322	1.84681905495019	0.00260985355445942	0.0269946226019193	0.0216122049483809	IL6/IL1B/STAT4/IL12RB1
REACTOME_PYROPTOSIS	25	0.63039573734784	1.93675836401405	0.00107381975398576	0.0142192615775037	0.0113841041587964	IL1A/IL1B/IRF1/BAK1/CASP4/CYCS/TP63/CHMP4B/IL18
REACTOME_PROGRAMMED_CELL_DEATH	191	0.356288705819984	1.59704620046667	0.000457089959077232	0.00739320000923577	0.00591908085474085	IL1A/IL1B/AVEN/BIRC3/IRF1/PSMD6/PSMC1/BCAP31/PSMD13/PSMD14/PSMB6/PSMC4/PSMD8/BID/PSMC2/DBNL/PSMB7/PSMB3/PSMB1/PSMB4/PSMB5/PSMA7/PSME3/PSMD12/PSMD2/BAK1/PSMA1/CASP4/PSMC5/PSMD4/DAPK3/CDH1/PMAIP1/PSMD3/PSMA5/YWHAQ/PSMA3/PSMD11/PSMB2/PSMF1/PSMA2/OMA1/DSG3/LMNA/PSMC3/PSMC6/OCLN/C1QBP/PSMA4/PSMD7/CYCS/HSP90AA1/UNC5B/STUB1/TP63/FADD/CHMP4B/IL18/PSMB10/STK24/PSMA6/DAPK2/CDC37
WP_OXIDATIVE_STRESS_RESPONSE	30	0.601541821039662	1.94574696830302	0.00101767559125042	0.0136255454161861	0.0109087681798948	CYP1A1/HMOX1/MAOA/NOX4/GPX3/UGT1A6/MGST1/TXNRD1/NQO1/TXN2/SOD1/NOX5/NFE2L2/MAPK10/GPX1/JUNB/XDH
WP_PHOTODYNAMIC_THERAPYINDUCED_NFKB_SURVIVAL_SIGNALING	31	0.769362889109306	2.50593754735177	5.27116095919563E-09	2.11724965194358E-06	1.69509439266765E-06	CXCL8/IL1A/IL6/CXCL2/MMP3/IL1B/MMP1/BIRC3/CCND1/NFKB2/ICAM1
BIOCARTA_NFKB_PATHWAY	21	0.519034166151175	1.52617701855322	0.0409492634325475	0.178458815320867	0.142876177542323	IL1A/MAP3K14/TNFAIP3

**FIGURE 3:**
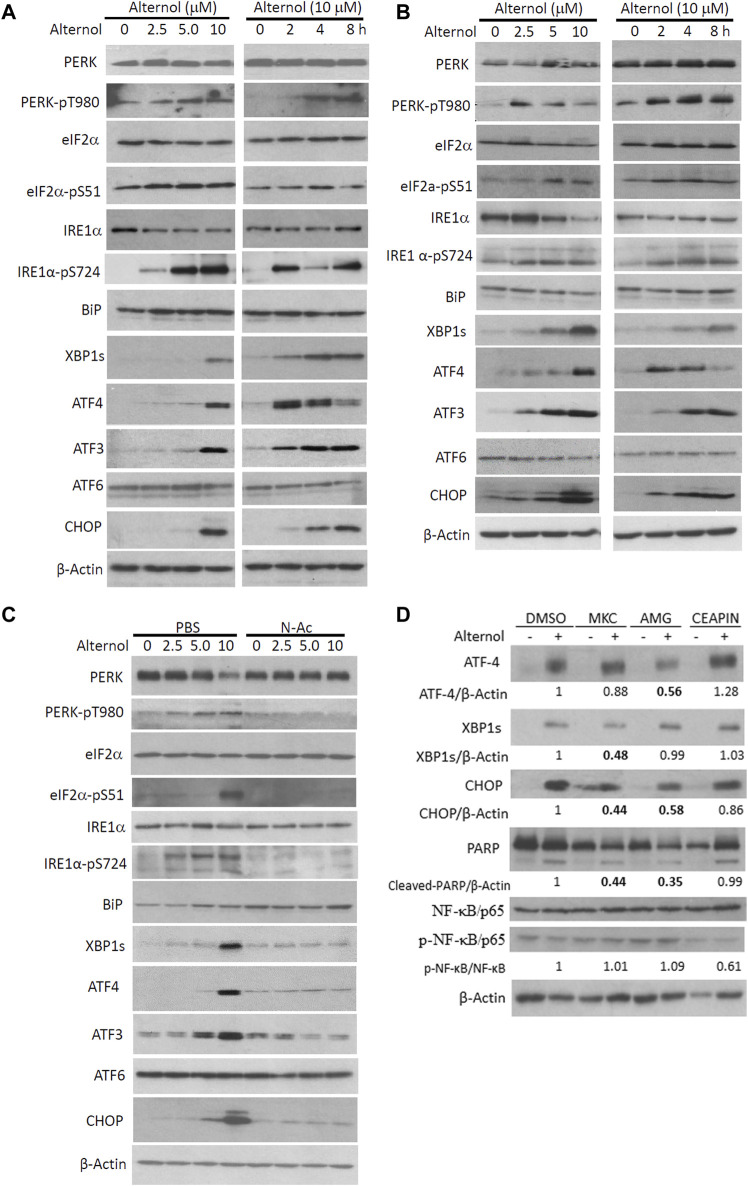
Alternol triggers ROS-dependent ER stress responses *via* PERK/IRE1α pathways. **(A,B)** PC-3 and 22RV1 cells were seeded in P100 dishes overnight and treated with Alternol at different concentrations or for the indicated period at 10 μM. **(C)** C4-2B cells were treated with Alternol at different concentrations as indicated with or without N-Ac (5 mM) for 6 h. **(D)** PC-3 cells were pre-treated with IRE1α inhibitor MKC8866 (10 µM), PERK inhibitor AMG44 (1 µM), and ATF6 inhibitor CEAPIN-A7 (10 µM) for 30 min followed by Alternol (10 µM) for 4 h. Equal amounts of cellular proteins were subjected to western blots with the antibodies as indicated.

It is well known that UPR and ER stress responses are monitored by three major cascades modulated by PERK, IRE1α, and ATF6 (Hetz et al., 2020). Activation of these pathways induces modifications on downstream effector proteins, leading to translational alteration and ER stress responses (Wiseman et al., 2022). We analyzed Alternol-induced changes of these ER stress-responding proteins (Sicari et al., 2020). Our results from three different prostate cancer cell lines showed that PERK and IRE1α were phosphorylated at their activating domain (PERK/T980 and IRE1α/S724) as early as 2 h after Alternol treatment (Figures 3A–C). Meanwhile, PERK downstream effector eIF2α protein was phosphorylated at the S51 site (Harding et al., 1999) and IRE1α/ATF6 downstream effector XBP1s protein level was also largely increased. In addition, the expression levels of classical ER stress-responding ATF4/ATF3/CHOP proteins were drastically increased after Alternol treatment in dose-dependent and time-dependent fashions. Consistent with our previous reports (Tang et al., 2014; Xu et al., 2019; Li et al., 2021), ROS scavenger N-Ac pretreatment abolished these alterations related to ER stress responses (Figure 3C). These data demonstrated that Alternol treatment induced strong UPR and ER stress responses *via* ROS-dependent mechanism (Yoshida et al., 2001).

We then asked which one or a combination of the three ER stress-responding pathways (Ron and Walter, 2007) were involved in Alternol treatment-induced ER stress. We utilized pathway-selective pharmacological inhibitors for these three pathways, IRE1α inhibitor MKC8866 (Sheng et al., 2019), PERK inhibitor AMG44 (Chintha et al., 2019), and ATF6 inhibitor CEAPIN-A7 (Xue et al., 2021) to determine their involvement in Alternol-induced ER stress response (Figure 3D). Our results showed that pretreatment with AMG44 reduced the Alternol-induced ATF4 expression while MKC8866 pretreatment suppressed Alternol-induced XBP1s expression. In addition, Alternol-induced CHOP protein expression was largely reduced by either AMG44 or MKC8866. Meantime, Alternol-induced PARP cleavage was also reduced by AMG44 or MKC8866. However, CEAPIN-A7 pre-treatment showed no significant attenuation on Alternol-induced these responses. These data suggest that both PERK and IRE1α cascades were involved in Alternol-induced UPR and ER stress.

### Alternol-induced ER stress is connected to immunogenic cell death-related ATP release

**FIGURE 4:**
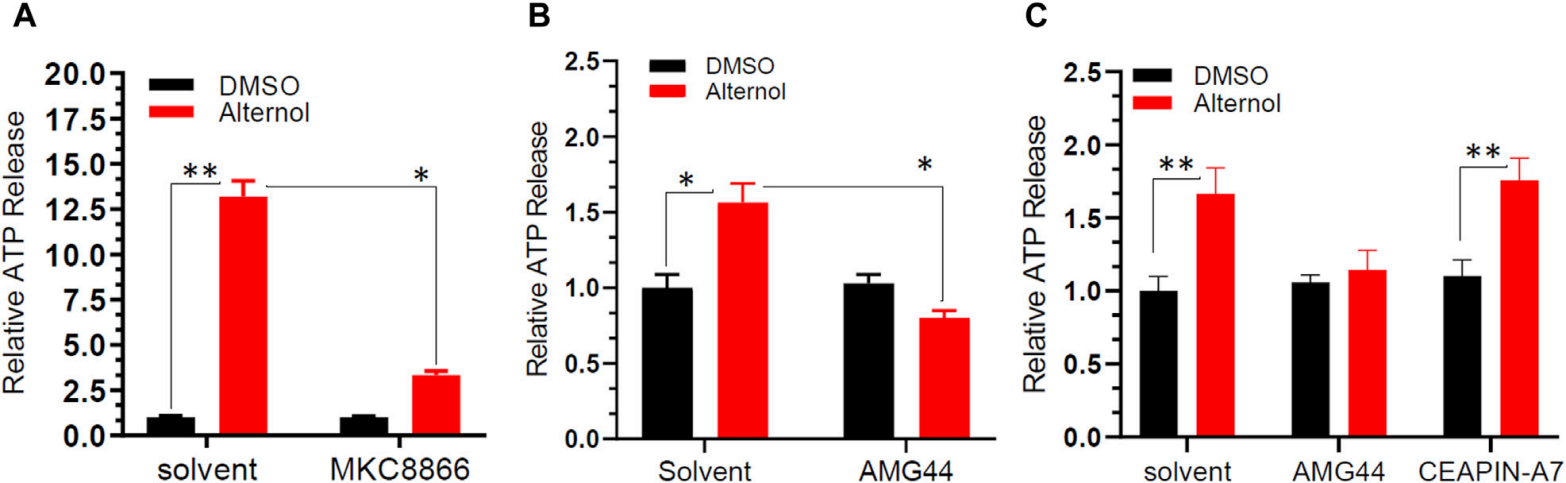
Alternol induces ATP release through PERK and IRE1α-dependent pathways. C4-2B panel **(A)** or PC-3 panel **(B,C)** cells were seeded in a 96-well plate overnight, and then pre-treated with the solvent, MKC8866 (10 μM), AMG44 (1 μM), or CEAPIN-A7 (10 μM) for 30 min, followed by Alternol (10 μM) for 6 h. The ATP level in the cell culture media was measured using the ATPlite Luminescence Assay System (catalog number 6016941, PerkinElmer). *p < 0.05; **p < 0.01, Student’s t-test.

Extracellular release of ATP molecules has been used as one of the hallmark indicators during immunogenic cell death under ER stress conditions (Galluzzi et al., 2020; Li et al., 2020; Liu et al., 2024). Because we recently demonstrated Alternol-elicited ATP molecule release during immunogenic cell death (Li et al., 2021), we then determined if Alternol-induced ER stress responses were accompanied by immunogenic ATP release. C4-2B cells were pre-treated with IRE1α inhibitor MKC8866, followed by Alternol treatment. As shown in Figure 4A, the Alternol treatment induced a drastic elevation of extracellular ATP level, which was significantly suppressed by MKC8866 pre-treatment. Similarly, PERK inhibitor AMG44 but not ATF-6α inhibitor CEAPIN-A7 suppressed Alternol-induced ATP release in PC-3 cells (Figures 4B, C). These data suggest that Alternol-induced ER stress response was related to immunogenic cell death elicited by Alternol *via* PERK and IRE1α cascades.

### PKR but not the NF-κB pathway is involved in alternol-induced ER stress responses

**FIGURE 5:**
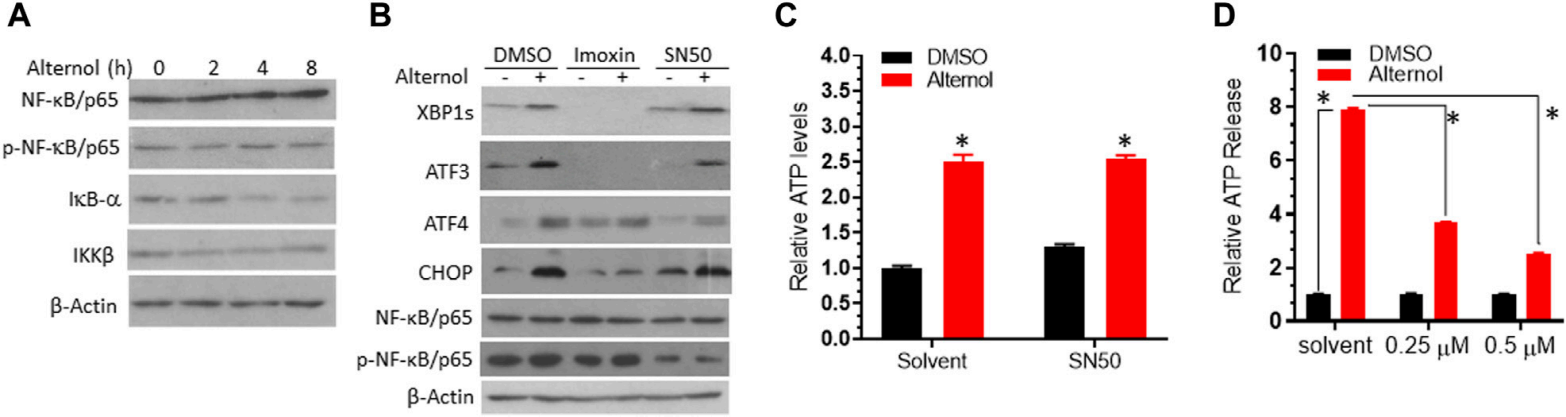
PKR but not the NF-κB pathway was involved in Alternol-induced ER stress. **(A)**. PC-3 cells were treated with Alternol (10 μM) for 0, 2, 4, 8 h. **(B)** C4-2B cells were pre-treated with Imoxin (10 μM) or SN50 (10 μM) for 30 min followed by Alternol (10 μM) for 6 h. Equal amounts of cellular proteins were subjected to western blots with the antibodies as indicated. β-Actin blots served as the protein loading control. **(C,D)** PC-3 cells were seeded in a 96-well plate overnight and then pre-treated with the solvent, SN50 (panel **C**), or Imoxin (panel **D**) for 30 min, followed by Alternol treatment for 6 h. ATP level in the cell culture media was measured using the ATPlite™ Luminescence Assay System. *, *p* < 0.05, Student’s t-test.

NF-κB pathway is a crucial regulator of inflammatory cytokine production (Capece et al., 2022), and oxidative stress is a common factor of NF-κB activation (Kim et al., 2001). Oxidative stress-induced NF-κB activation has been implicated in immunogenic cell death (Zhao et al., 2022). We then asked if Alternol treatment activated the NF-κB pathway. We first re-analyzed the RNA-seq data with the GSEA approach. As expected, GSEA analysis revealed that the NF-κB pathway was enriched in Alternol-induced activation of gene expression (Table 2). We then evaluated the changes in major modulators of NF-κB activation including NF-κB/p65, IκBα, and IKKβ proteins. As shown in Figure 5A, Alternol treatment reduced the protein levels of IκB-α, the negative regulator of NF-κB activation, in a time-dependent manner. Next, we examined if NF-κB inhibition suppressed Alternol-induced ER stress responses and ATP release. Unexpectedly, pretreatment with NF-κB inhibitor SN50 (Lin et al., 1995) had no significant suppression on Alternol-induced elevation of XBP1s and CHOP protein levels (Figure 5B), as well ATP release in PC-3 cells (Figure 5C), although SN50 slightly reduced ATF4 expression. As expected, SN50 largely reduced the phosphorylation level of NF-κB/p65, confirming the SN50 action (Wu et al., 2020). In addition, pretreatment with IRE1α inhibitor MKC8866, PERK inhibitor AMG44, and ATF6 inhibitor CEAPIN-A7 all had no significant effect on NF-kB phosphorylation (Figure 3D). These data indicate that NF-κB activation is not a major player in Alternol-induced ER stress responses and ATP release.

Lastly, we evaluated the involvement of protein kinase R (PKR) in Alternol-induced ER stress response, since PKR was recently reported to modulate ER stress-related induction of ATF3, CHOP, and XBP1s expression (Guerra et al., 2006; Eo and Valentine, 2022). A PKR-specific inhibitor Imoxin (Nakamura et al., 2014) was utilized as a pretreatment during Alternol-induced ER stress. As shown in Figure 5B, Imoxin pretreatment largely reduced the protein levels of XBP1s and ATF3 at the basal and Alternol treatment conditions. Meanwhile, Imoxin also blunted the Alternol-induced increase of ATF4 and CHOP proteins. In addition, Imoxin significantly suppressed Alternol-induced ATP release in a concentration-dependent manner (Figure 5D). These data strongly suggest that PKR activation was involved in Alternol-induced ER stress response, leading to immunogenic cell death.

## Discussion

In this study, we demonstrated that Alternol interacted with multiple mitochondrial and ER chaperone proteins and elicited ROS-dependent ER stress responses in prostate cancer cells. Alternol-induced ER stress responses involved three protein kinases, PKR, PERK, and IRE1α, resulting in eIF2α phosphorylation, XBP1s processing, ATF3/ATF4 overexpression, and CHOP protein accumulation. Inhibition of these cascades suppressed immunogenic ATP release. According to the results, we proposed that Alternol induces immunogenic cell death *via* ER stress-related cascades of PKR, PERK, and IRE1α kinases.

Alternol is a novel small molecular compound and preclinical studies from our group and others have shown its potency in specifically killing multiple types of human cancer cells *via* ROS-dependent mechanism (Liu et al., 2020). Most interestingly, our recent studies discovered that Alternol-induced cancer cell killing elicited a strong immunogenic response that resulted in xenograft tumor suppression in immune-intact mice (Li et al., 2021). Consistent with the notion that ER stress response is crucial in DAMP release and immunogenic elicitation (Aria and Rezaei, 2023), in this study, our data confirmed the ER stress responses after Alternol treatment in prostate cancer cells. Our studies discovered that three ER stress-related protein kinases, PKR, PERK, and IRE1a, were involved in Alternol-induced ER stress responses and immunogenic ATP release. Our results also verified our previous report (Li et al., 2019) that Alternol interacted with five chaperone proteins resided in mitochondria and ER and Alternol treatment increased their expression levels, a potential response due to the UPR and ER stress.

It is well known that there are three sensor kinases, IRE1α, PERK, and ATF-6, responding to ER stress conditions (Hetz et al., 2020; Sicari et al., 2020). IRE1α induces the unconventional splicing of XBP1 mRNA to produce a shorter XBP1s protein, PERK kinase induces eIF2α phosphorylation at serine 51 to inactivate protein translation, and ATF6 N-terminal region exerts a transcriptional activity to upregulate UPR-related gene after undergoing proteolytic cleavage (Hetz et al., 2020; Saaoud et al., 2024). In this study, our data revealed that PERK and IRE1α but not ATF6 cascades were involved in Alternol-induced ER stress. ATF6 protein did not show a proteolytic change and its specific inhibitor failed to suppress XBP1 expression and immunogenic ATP release after Alternol treatment. A further mechanistic study is warranted to dissect Alternol-induced activation of PERK and IRE1α cascades, although ROS dependency was confirmed (Li et al., 2021; Yang et al., 2024).

PKR is one of the four eIF2α kinases (Jackson et al., 2010) and it is mainly activated after viral infection in mammalian cells (Park et al., 2006; Zhang and Karijolich, 2024). However, recent studies showed that PKR activation was also involved in eIF2α/S51 phosphorylation and immunogenic cell death induced by chemo-drugs in melanoma and breast cancer cells (Giglio et al., 2018; Li et al., 2022). In addition, PKR-specific inhibitor Imoxin suppressed saturated fatty acid-induced ER stress responses including XBP1s processing, ATF6, and CHOP expression (Eo and Valentine, 2022). Interestingly, we also found that Imoxin pretreatment almost blunted Alternol-induced XBP1s, largely reduced ATF4 and CHOP expression, and significantly reduced immunogenic ATP release, indicating a signaling crosstalk among the conventional ER stress sensors and PKR during Alternol-induced ROS-dependent immunogenic response.

**FIGURE 6:**
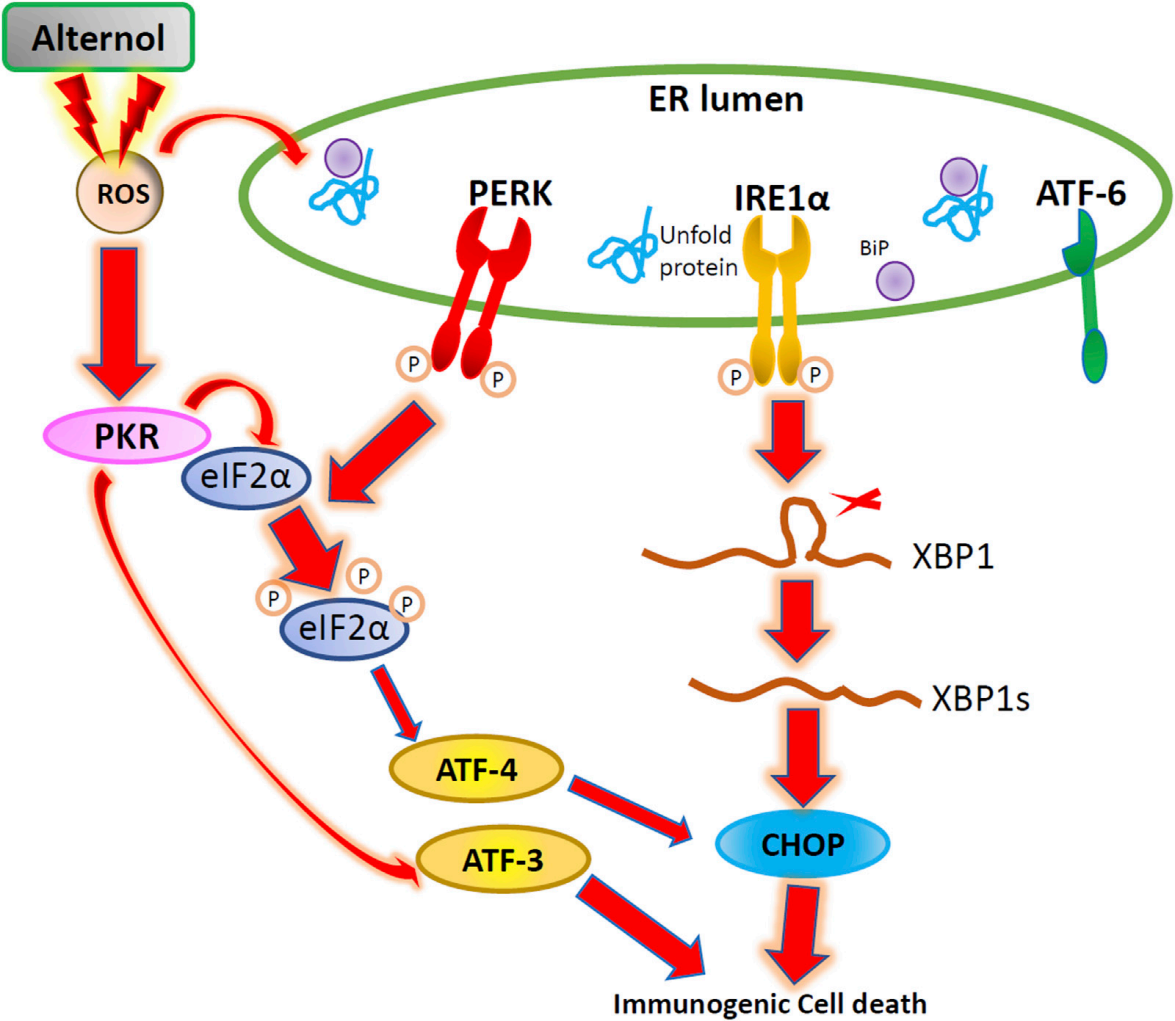
The Schematic drawing for Alternol-induced ER stress in prostate cancer. Alternol treatment causes ROS accumulation, leading to PKR, PERK, and IRE1α activation and subsequent eIF2α phosphorylation, ATF3/ATF4 transactivation, XBP1 splicing, and CHOP expression.

In conclusion, we demonstrated that Alternol treatment triggered ROS-dependent ER stress responses, linking to immunogenic ATP release. We also proved that Alternol-induced ER stress involved three protein kinases, IRE1α, PERK, and PKR, but not ATF6 protein (Figure 6). Further mechanistic investigation is needed to dissect the crosstalk among these three kinase cascades under oxidative stress.

## Data Availability

The datasets presented in this study can be found in online repositories. The names of the repository/repositories and accession number(s) can be found below: http://www.ncbi.nlm.nih.gov/bioproject/705723.
